# Supplementary figures and images for: Effects of rhodomyrtone on Gram-positive bacterial tubulin homologue FtsZ
Source: PeerJ. 2017 Feb 2;5:e2962. doi: 10.7717/peerj.2962 (PMC5292029; doi:10.7717/peerj.2962)

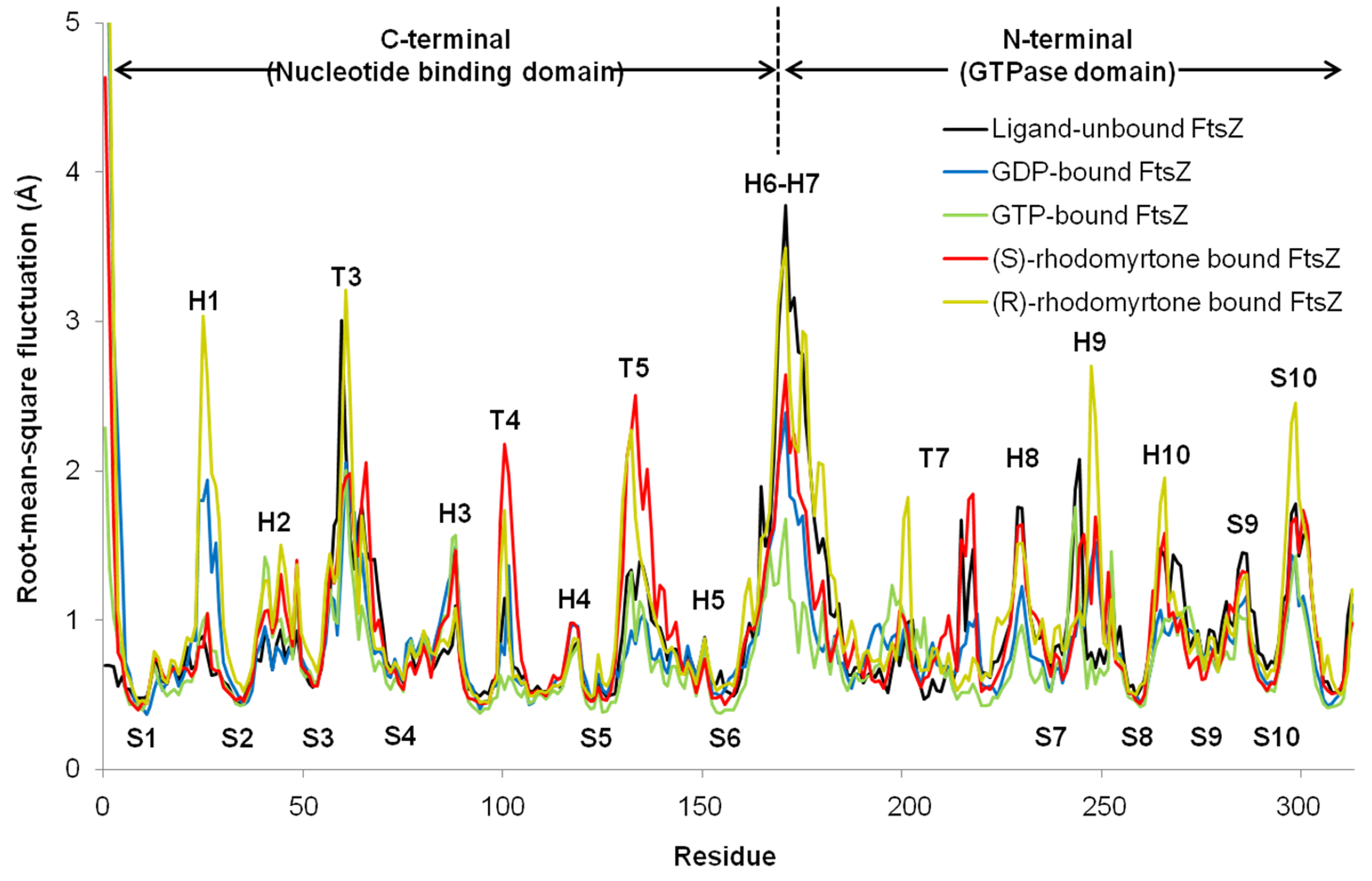

Supplement: Figure S1 — Black for ligand-free FtsZ, blue for GTP-FtsZ, green for GDP-FtsZ, red for (S)-rhodomyrtone-FtsZ, and yellow for (R)-rhodomyrtone. The regions with the most significant changes are labeled. [file peerj-05-2962-s001.png]
