# Supplementary material for: Effects of rhodomyrtone on Gram-positive bacterial tubulin homologue FtsZ
Source: PeerJ. 2017 Feb 2;5:e2962. doi: 10.7717/peerj.2962 (PMC5292029; doi:10.7717/peerj.2962)
Supplement: Data S3 [file peerj-05-2962-s004.docx]

**Sukanlaya Leejae^1^, Peter William Taylor^2^, and Supayang Piyawan Voravuthikunchai^1^**

^1^Department of Microbiology and Natural Products Research Center, Faculty of Science,

Prince of Songkla University, Songkhla 90112, Thailand

^2^School of Pharmacy, University College London, London WC1N 1AX, UK

**Method**


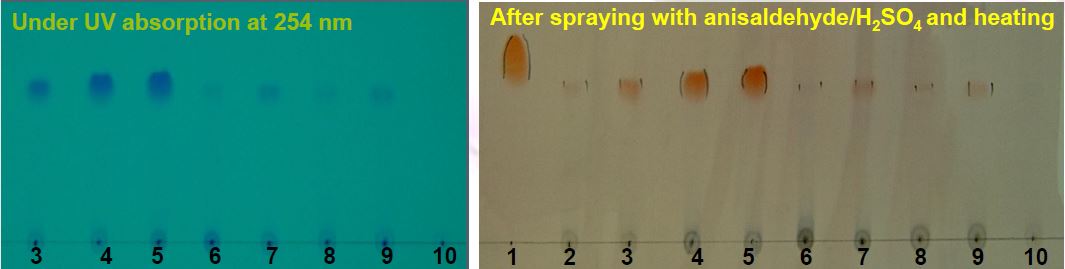
Thin-layer chromatography (*Leejae et al., 2013*).

**Results**

**Figure:** Localization of rhodomyrtone in *S. aureus* ATCC 29213 after treated with 8 μg/ml of the compound for 1-4 h. Lanes: 1, reference rhodomyrtone; 2 and 6, rhodomyrtone-treated *S. aureus* for 1 h; 3 and 7, rhodomyrtone-treated *S. aureus* for 2 h; 4 and 8, rhodomyrtone-treated *S. aureus* for 3 h; 5 and 9, rhodomyrtone-treated *S. aureus* for 4 h; 10: 1% DMSO-treated *S. aureus* for 4 h; 2-5, cell wall and cell membrane fractions; 6-9, cytoplasmic fraction.

**Reference**

**Leejae S, Taylor PW, and Voravuthikunchai SP. 2013.** Antibacterial mechanisms of rhodomyrtone against important hospital-acquired antibiotic-resistant pathogenic bacteria. *Journal of Medical Microbiology* **62(1)**:78-85. DOI 10.1099/jmm.0.049205-0.
